# Understanding the Deactivation Phenomena of Small-Pore Mo/H-SSZ-13 during Methane Dehydroaromatisation

**DOI:** 10.3390/molecules25215048

**Published:** 2020-10-30

**Authors:** Miren Agote-Arán, Anna B. Kroner, David S. Wragg, Wojciech A. Sławiński, Martha Briceno, Husn U. Islam, Igor V. Sazanovich, María E. Rivas, Andrew W. J. Smith, Paul Collier, Inés Lezcano-González, Andrew M. Beale

**Affiliations:** 1 Chemistry Department, University College of London Gordon Street, London WC1H 0AJ, UK; uccamag@ucl.ac.uk; 2 Diamond Light Source Ltd., Harwell Science and Innovation Campus, Didcot OX11 0DEU, UK; anna.kroner@diamond.ac.uk; 3 Department of Chemistry, NGAP Centre for Research Based Innovation, University of Oslo, N-0315 Oslo, Norway; david.wragg@kjemi.uio.no (D.S.W.); wslawinski@chem.uw.edu.pl (W.A.S.); 4 Faculty of Physics, University of Warsaw, Pasteura 1 Street, 02-093 Warsaw, Poland; 5 Johnson Matthey Technology Centre, Blount’s Court, Sonning Common, Reading RG4 9NH, UK; BriceM01@matthey.com (M.B.); Husn.Islam@matthey.com (H.U.I.); maria.rivas-velazco@matthey.com (M.E.R.); andrew.smith@matthey.com (A.W.J.S.); collip02@matthey.com (P.C.); 6 Central Laser Facility, Research Complex at Harwell, Science and Technology Facilities Council, Harwell Campus, Didcot OX11 0QX, UK; igor.sazanovich@stfc.ac.uk; 7 Research Complex at Harwell, STFC Rutherford Appleton Laboratory, Didcot OX11 0FA, UK

**Keywords:** MDA, Mo/CHA, *operando* XAS/XRD

## Abstract

Small pore zeolites have shown great potential in a number of catalytic reactions. While Mo-containing medium pore zeolites have been widely studied for methane dehydroaromatisation (MDA), the use of small pore supports has drawn limited attention due to the fast deactivation of the catalyst. This work investigates the structure of the small pore Mo/H-SSZ-13 during catalyst preparation and reaction by operando X-ray absorption spectroscopy (XAS), in situ synchrotron powder diffraction (SPD), and electron microscopy; then, the results are compared with the medium pore Mo/H-ZSM-5. While SPD suggests that during catalyst preparation, part of the MoO_x_ anchors inside the pores, Mo dispersion and subsequent ion exchange was less effective in the small pore catalyst, resulting in the formation of mesopores and Al_2_(M_O_O_4_)_3_ particles. Unlike Mo/H-ZSM-5, part of the Mo species in Mo/H-SSZ-13 undergoes full reduction to Mo^0^ during MDA, whereas characterisation of the spent catalyst indicates that differences also exist in the nature of the formed carbon deposits. Hence, the different Mo speciation and the low performance on small pore zeolites can be attributed to mesopores formation during calcination and the ineffective ion exchange into well dispersed Mo-oxo sites. The results open the scope for the optimisation of synthetic routes to explore the potential of small pore topologies.

## 1. Introduction

Methane dehydroaromatisation (MDA) reaction converts methane directly into aromatics and light hydrocarbons, giving H_2_ as a co-product. The route has a great potential for the efficient valorisation of natural gas, but its commercialisation is impeded by the rapid catalyst deactivation due to carbon deposit formation. Since the first report on MDA by Wang et al. [1], most of the catalysts studied for this reaction comprise Mo supported in medium pore H-ZSM-5 zeolite with MFI structure [2]. The extensive work performed in Mo/H-ZSM-5 include the optimisation of synthetic procedures [3,4,5,6], modelling studies [7,8,9,10], the development of engineering solutions [11,12,13], and structural investigations to study the active role of Mo, zeolite, or carbon deposits [14,15,16,17,18,19,20,21,22,23,24,25,26,27]. While other zeolite topologies have been investigated, these studies often focus in medium pore zeolites mostly because the dimensions of such pores are believed to provide shape selectivity to benzene, which comprises up to 80% of the MDA products [28,29]. Nonetheless, good catalytic performance and significant yield to aromatics have been also reported for non-microporous supports [30]. Recent structural studies point out that the evolution of Mo speciation—from isolated Mo-oxo species to carburised Mo clusters—under reaction conditions is also an important factor in MDA product distribution [15,20,21]. Furthermore, the reported migration of Mo species from H-ZSM-5 pores to the outer surface during MDA is likely to restrain the shape selective effects of the zeolite [14,20,31].

In the recent years, small pore zeolites have received great attention in a number of catalytic reactions; such structures allow tuning the product selectivity while providing high thermal stability. In the case of MDA, the use of small pores could potentially increase selectivity to C_2_ molecules such as ethane or ethylene, which are also intermediates of the reaction. Compared to aromatics, light hydrocarbons are of greater importance for the chemical industry, as they are the precursors of a range of polymers (i.e., polyvinyl chloride, polyethylene), solvents, surfactants, or anaesthetic agents. A better thermal stability is also advantageous for MDA, which occurs at >650 °C. In addition, it has been suggested that zeolites with relatively large cages but small pores could restrict the sintering of supported metal clusters, which would be beneficial to prevent Mo-based catalyst deactivation. Under reaction conditions, growing metal particles can become entrapped in the zeolite once the size of the particle exceeds the diameter of the cage window, and further sintering and migration is prevented [32]. Nonetheless, very few studies have been reported using small pore zeolites for methane activation in non-oxidative conditions. Zhang et al. reported 0.7% methane conversion when using small pore Mo/SAPO-34 with a CHA structure, which is one order of magnitude lower than for the Mo/H-ZSM-5 values obtained in the same study [33]. The selectivity to benzene (calculated excluding coke formation) was lower for the small pore (73%) than for the medium pore (91%). Consistent with the pore dimensions, higher selectivity to small C_2_ molecules was achieved on Mo/SAPO-34 (10%) compared with Mo/H-ZSM-5 (4.5%). A recent publication on Mo/H-SSZ-13, also with CHA structure [34], reported low conversions (≈0.7%) and practically no aromatics formation with most of the methane being converted to carbon deposits. The high selectivity to carbon deposits on Mo/CHA was attributed to the inability of benzene to leave the small CHA pore openings, resulting in an increased selectivity to carbon deposits. However, up to date, no detailed structural studies have been reported on small pore materials to account for their catalytic performance.

The MFI topology comprises a three-dimensional channel arrangement with two 10 ring channel systems [35,36]: straight channels running parallel to (010) with a pore diameter ≈5.3 × 5.5 Å and interconnected to these, sinusoidal channels parallel to (100) of ≈5.1 × 5.4 Å diameter. As such, the MFI structure presents no cages or cavities. The CHA framework also presents a three-dimensional channel system [37,38]; it is composed of 6-membered ring pores arranged in an AABBCC sequence. This stacking comprises double 6-membered ring (D6R) units connected to ellipsoidal large cages of 6.7 × 10 Å, which results in 8-membered ring (8R) windows of 3.8 × 3.8 Å. Considering the kinetic diameter of different molecules involved in MDA [39,40], the pores in the CHA structure are too small for aromatic molecules to diffuse through; if zeolite topologies plays a role in product distribution, an increased selectivity to light hydrocarbons would be expected. On the other hand, the cages are large enough to host benzene and toluene, which could lead to carbon deposit formation. Yet, groups that investigated MCM-22 zeolite for dehydroaromatisation claim that the longer catalyst lifetime obtained with this zeolite is in fact due to its large cage system [29,41]. They propose that the cages preferentially accommodate carbon deposits, leaving the rest of the pore and channel system free for molecule circulation.

The present work studies the properties of small pore Mo/H-SSZ-13 compared with the widely studied medium pore Mo/H-ZSM-5, using comparable Mo loadings (≈4 wt %) and Si/Al ratios (of 14 to 17). The location of Mo after catalyst preparation is studied by synchrotron powder diffraction, while its evolution during a series of reaction—regeneration cycles is investigated by *operando* X-ray absorption and supported by further characterisation such as microscopy, thermogravimetric analysis, and Raman spectroscopy.

## 2. Results

### 2.1. Study of the Calcination Process

The location of Mo on H-SSZ-13 during calcination was studied by in situ synchrotron powder diffraction (SPD). X-ray diffraction data were collected during calcination of the as-prepared catalyst (up to 600 °C, 6 °C/min under 20% O_2_/He). The observed, calculated, and difference patterns obtained from Rietveld refinement are given in Figure S1 in the electronic supporting information (ESI); the experimental parameters and goodness-of-fit factors are given in Table S1, while the atomic coordinates and selected bond distances and angles can be found in Tables S2–S4. The non-framework electron density observed in difference Fourier maps is assumed to be due to water (below about 200 °C) and Mo (above 400 °C, after the MoO_3_ precursor peaks began to disappear from the diffraction patterns). This was fitted using dummy atoms as described by Wragg et al. [42].

**Figure 1:**
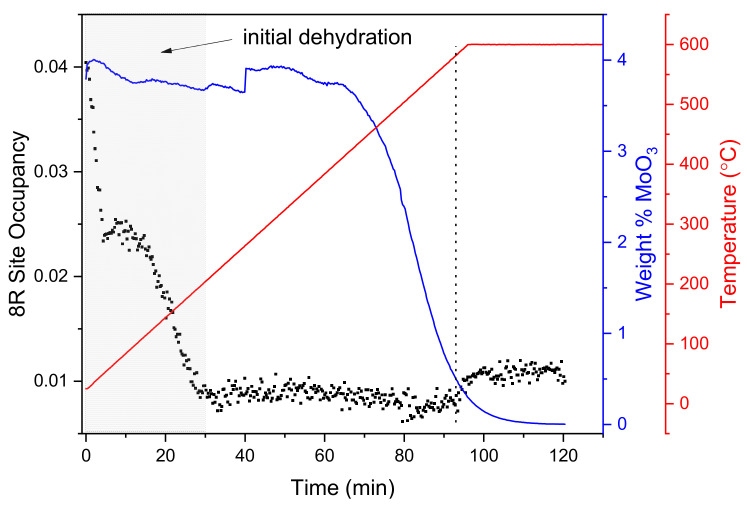
Synchrotron powder diffraction (SPD) results for MoO_3_ and H-SSZ-13 physical mixture showing the Mo occupancy in the 8-membered ring (8R) position of the CHA structure (black dots) during calcination (room temperature to 600 °C in 20% O_2_/He). The wt % of crystalline MoO_3_ precursor (blue line) during calcination and the sample temperature (red line) are indicated in the right axes. The region of the experiment in which water is leaving the zeolite framework (up to ≈200 °C) is indicated by grey shading, the dotted vertical line indicates the point where the 8R site occupancy begins to increase as the wt % of MoO_3_ decreases.

The Rietveld analysis results for the calcination process of the SSZ-13 and MoO_3_ physical mixture are summarised in Figure 1. As the temperature increases up to 200 °C, zeolite dehydration takes place, and the electron density in the 8-membered rings (8R) of the CHA structure (left axis) drops drastically as a result of water removal from the framework. As calcination advances, the MoO_3_ precursor crystals start to disappear around 400 °C (right axis). According to previous diffraction studies [25,43], this loss of precursor crystallinity is attributed to the migration of MoO_x_ into the zeolite micropores. As the weight percentage of MoO_3_ drops, the electron density at the 8R site increases slightly (≈570 °C), suggesting that some Mo occupies these sites. The increase could be ascribed to the ion exchange at the zeolite Brønsted acid sites, with the attachment of Mo in the vicinity of 8R. This hypothesis is further supported by studies performed by Borry et al., who measured the H_2_O evolution rate by mass spectrometry during the calcination of MoO_3_ and H-ZSM-5 mixtures and reported ion exchange and anchoring of Mo to occur within this temperature range [25]. The observed increase in electron density at the 8R site is small and occurs when much of the MoO_3_ has already disappeared, suggesting that some Mo remains outside the zeolite pores after calcination, as described in the earlier studies mentioned above.

**Figure 2:**
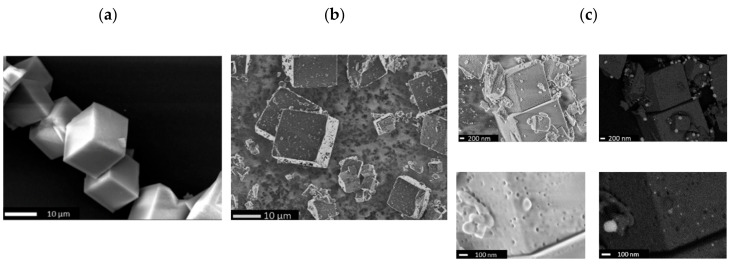
SEM images acquired for (**a**) parent zeolite H-SSZ-13, (**b**) Mo/H-SSZ-13 after calcination in air (700 °C, 5 °C/min, 30 min), and (**c**) comparison of secondary and backscattering electron images at different magnifications for calcined Mo/H-SSZ-13.

The high-resolution secondary electron images for the parent H-SSZ-13 (Figure 2a) show ≈10 µm crystals with cubic morphology. The images taken for Mo/H-SSZ-13 after calcination (Figure 2b) exhibit defects as well as smaller particles likely arising as a result of the mechanical destruction during the grinding step of the synthesis (i.e., 30 min of mixing of zeolite and MoO_3_ in a mortar). High-magnification secondary electron images of calcined Mo/H-SSZ-13 (Figure 2c) reveal the presence of large mesopores (up to 50 nm) on the zeolite; the corresponding backscattered electron images show bright particles on the zeolite surface corresponding to molybdenum-rich particles of varying sizes (up to 100 nm). These observations indicate that MoO_3_ reacts with the zeolite during calcination, resulting in the extraction of framework Al and the formation of Al_2_(MoO_4_)_3_ particles, which is a phenomena also reported for Mo/H-ZSM-5 [44]. This was corroborated by transmission electron microscopy combined with energy dispersive X-ray elemental analysis (TEM-EDX) which showed the particles on the zeolite surface to be composed of Mo and Al (see Figure S3); however, we note that the EDX spectra taken in different regions of the crystals confirm the dispersion of part of Mo in the H-SSZ-13 despite the formation of Al_2_(MoO_4_)_3_ particles.

### 2.2. Operando X-Ray Absorption Spectroscopy

The Mo evolution during MDA reaction was followed by *operando* X-ray absorption spectroscopy (XAS). In a first experiment, the as-prepared samples (MoO_3_ and zeolite physical mixture) were calcined at 700 °C for 30 min under 20% O_2_/He. Then, the MDA reaction was performed by flowing 50% CH_4_/Ar at 700 °C for 90 min (GHSV = 3000 h^−1^). During the reaction, XAS spectra were continuously collected while the gas composition in the outlet was measured by online mass spectrometry (MS).

**Figure 3:**
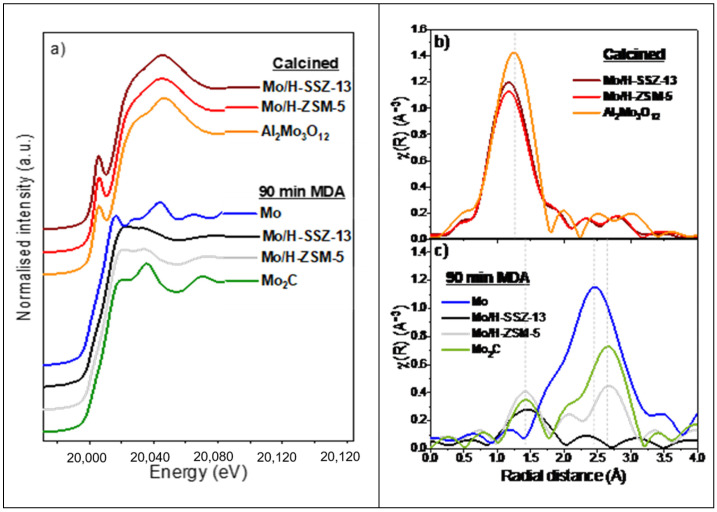
Mo K-edge XAS spectra showing: (**a**) XANES of Mo/zeolites after calcination and after 90 min of methane dehydroaromatisation (MDA) reaction (700 °C, 50% CH_4_/Ar, gas hour space velocity (GHSV) = 300 h^−1^) together with Al_2_(MoO_4_)_3_, Mo, and Mo_2_C references; (**b**) FT-EXAFS of calcined Mo/zeolite compared to Al_2_(MoO_4_)_3_; and (**c**) FT-EXAFS of Mo/zeolites after 90 min of MDA compared to Mo_2_C and metallic Mo. The maxima in the FT-EXAFS of the reference spectra is indicated by vertical dashed lines.

To clearly visualise differences in metal speciation in Mo/H-SSZ-13 and Mo/H-ZSM-5, Figure 3 compares the Mo K-edge XAS spectra before and after reaction at 700 °C; the figure also includes reference spectra for Al_2_(MoO_4_)_3_, Mo_2_C, and metallic Mo. The full set of XAS data collected during reaction can be found in Figure S4 in the ESI, together with the MS trends which verify that the species observed correspond to the Mo centres present under MDA conditions.

The X-ray absorption near edge structure (XANES) of the calcined Mo/H-ZSM-5 and Mo/H-SSZ-13 were comparable (Figure 3a); both catalysts show an adsorption edge at ≈20014 eV (1s → 5p transition) and an intense pre-edge peak at 20,005 eV (1s → 4d quadrupole transition) [26]. These features were analogous to the Al_2_(MoO_4_)_3_ reference, which contains isolated tetrahedral MoO_4_ units. The Fourier transform X-ray absorption fine structures (FT-EXAFS) shown in Figure 3b exhibit similar features with a main peak around 1.3 Å corresponding to scattering from near neighbour O atoms. According to previous reports, the ion-exchanged Mo species on the zeolites corresponds to Mo-oxo centres in tetrahedral environment comprising two bridging Mo-O bonds and two shorter terminal Mo=O bonds [16,20,26]. Compared with Al_2_(MoO_4_)_3_, both Mo/zeolite samples exhibit a lower intensity of the O scattering peak as well as a shift to lower radial distances (1.2 Å instead of 1.3 Å); these features are consistent with the presence of ion exchanged Mo-oxo species with shorter Mo=O bonds. In the case of Mo/H-SSZ-13, we expect a contribution of Al_2_(MoO_4_)_3_, which—as described above—arises due to the strong zeolite dealumination taking place during the calcination step. Ion exchanged Mo-oxo species show a similar tetrahedral local structure to Al_2_(MoO_4_)_3_, resulting in comparable spectra that makes the quantification of these two species difficult by XAS.

After 90 min of reaction, both Mo/H-SSZ-13 and Mo/H-ZSM-5 show XANES spectra similar to Mo_2_C implying a carburisation of molybdenum species, as reported previously [2,29]. The FT-EXAFS of Mo/H-ZSM-5 in Figure 3c comprises two peaks at 1.4 and 2.7 Å consistent with scattering from C and Mo neighbours in Mo_2_C reference. Interestingly, the FT-EXAFS of Mo/H-SSZ-13 reveals no contribution at 2.7 Å. A close inspection of the EXAFS (Figure S6b in the ESI) shows that while both catalysts exhibit oscillations comparable to Mo_2_C, Mo/H-SSZ-13 presents additional features that coincide with metallic Mo (Mo^0^). The absence of a Mo-Mo scattering peak in the Mo/H-SSZ-13 FT-EXAFS is attributed to the destructive interaction between Mo_2_C and Mo^0^ EXAFS oscillations. These results point out that part of Mo centres in H-SSZ-13 undergo full reduction to Mo^0^ under MDA conditions, which has not been previously observed in other topologies.

In a second experiment, the regeneration of Mo/H-SSZ-13 was studied by performing three consecutive reaction–regeneration cycles. The cycles consisted of an initial calcination at 700 °C in air (cycle 1, calc.) followed by MDA at 650 °C (cycle 1, MDA 650 °C). Then, the catalyst was regenerated by burning off the coke with 20% O_2_/He flow (cycle 2, calc.). After a second MDA reaction at 650 °C (cycle 2, MDA 650 °C) and consequent regeneration (cycle 3, calc.), a last MDA reaction was performed at 780 °C (cycle 3, MDA 780 °C). The MDA cycles were 70 min long, while regenerations with O_2_ were performed for 10 min.

**Figure 4:**
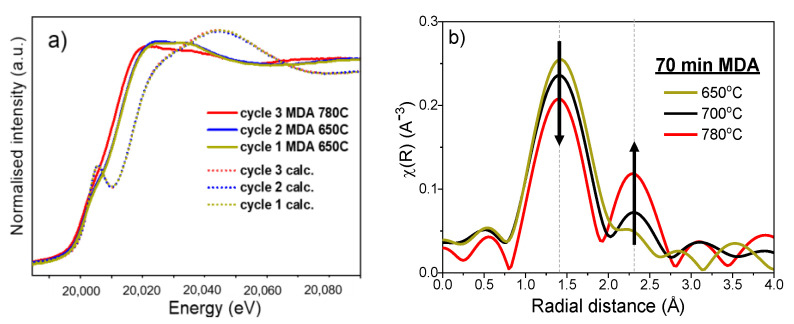
(**a**) Mo K-edge XANES obtained of Mo/H-SSZ-13 during the consecutive calcination and MDA cycles, and (**b**) Mo K-edge FT-EXAFS of Mo/H-SSZ-13 after 70 min of MDA reaction at 650 (cycle 1), 700, and 780 °C (cycle 3).

Figure 4a shows the Mo K-edge XANES spectra after each calcination and MDA reaction step. All the spectra after oxygen treatment are identical, indicating the reoxidation of the carburised Mo. Such regeneration is possibly due to the oxidation of Mo carbides to volatile MoO_3_, which diffuses back into the zeolite pores, resulting in ion exchange to form Mo-oxo sites; of course, we cannot rule out that during regenerations, Al_2_(MoO_4_)_3_ is formed again due to reaction with extraframework alumina. Regarding the reaction cycles, the spectra after the third MDA at 780 °C shows an absorption edge at lower energies than the cycles at 650 °C; this indicates a higher degree of Mo reduction at increased MDA temperatures. To gain insight into the effect of reaction temperature, Figure 4b compares the FT-EXAFS features after 70 min of MDA performed at 700 °C in the first experiment with the cycles performed at 650 °C (Cycle 1) and 780 °C (Cycle 3). The figure shows a decreased intensity of Mo-C scattering peak (1.4 Å) with increasing temperature, which is accompanied with an increasing contribution of the Mo-Mo scattering path in Mo^0^ (≈2.3 Å). This was also supported by inspection of EXAFS (Figure S6c) where the features corresponding to Mo^0^ became increasingly pronounced with the temperature.

The integer Mo oxidation state of Mo/H-SSZ-13 was evaluated during the two experiments described above (700 °C MDA, and the consecutive reaction-regeneration cycles at 650 to 780 °C). This analysis was carried out by following the same procedure reported in previous works [15,20] where the correlation between the edge position—at the half-step height of the absorption edge—and oxidation state was obtained by a linear fit using references with known oxidation states (MoO_2_, MoO_3_, and Mo_2_C). As listed in Table 1, the Mo oxidation state of the as-prepared sample was 5.9, which is consistent with the MoO_3_ precursor with Mo^6+^. After each calcination cycle, the oxidation Mo also appears in oxidised form, exhibiting an integral oxidation state of 5.8; as discussed above, this would correspond to a mixture of species (i.e., ion exchange Mo-oxo and Al_2_(MoO_4_)_3_. After 70 min of MDA, oxidation states of 2.4, 2.1, and 1.8 were obtained for reactions performed at 650, 700, and 780 °C cycles respectively, which is in line with the increased degree of Mo reduction with the reaction temperature.

Table 1 also lists the Mo K-edge absorption edge steps obtained in the consecutive cycles. The edge step exhibits a gradual drop over the course of the experiment and particularly after each calcination, suggesting that Mo concentration in the sample (field of view) decreases as a result of the sublimation of Mo oxide species.

### 2.3. Laboratory MDA Investigations

**Figure 5:**
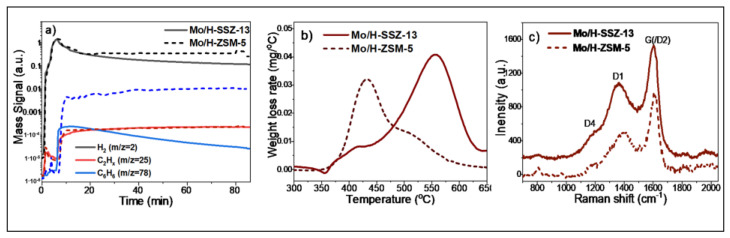
(**a**) MS data under methane dehydroaromatisation reaction products collected for Mo/H-SSZ-13 and Mo/H-ZSM-5 (50% CH_4_/Ar, 1500 h^−1^, 700 °C, 90 min); the data are shown in logarithmic scale, and all signals are normalised to the carrier gas signal (Ar); (**b**) The derivative of the TGA curves for the catalysts recovered after 90 min of reaction; and (**c**) Raman spectra for the catalysts recovered after 90 min of reaction.

The elemental analysis of calcined Mo/H-SSZ-13 and Mo/H-ZSM-5 (Table S5) indicated similar Mo contents of 3.9 and 3.8 wt % respectively, and Si/Al ratios of 14.0 and 16.8. The activity of both catalysts was compared by performing the MDA reaction for 90 min (50% CH_4_/Ar, GHSV = 1500 h^−1^, 700 °C). The MS data collected are shown in Figure 5a. For simplicity, only the mass trends corresponding to the main reaction products are presented (i.e., H_2_, C_2_H_x_, and C_6_H_6_), plots containing all recorded mass trends can be found in Figure S5 in the ESI. Both catalysts exhibit an induction period in which H_2_ is formed but no hydrocarbon production is detected; CO, CO_2_, and H_2_O evolution is also seen in the induction period (shown in Figure S5). It is widely accepted that during this stage, oxidic Mo species undergo reduction and carburisation, which is in line with the XAS data shown in Figure 3 and Figure 4. After ≈ 7 min, the induction period gives way to the aromatisation stage, wherein C_2_H_x,_ H_2_ and aromatic (i.e., C_6_H_6_) formation is seen. While normalised C_2_H_x_ signal intensities in both catalysts are comparable, the C_6_H_6_ detection is noticeably weaker in the small pore catalysts. This suggests that unlike the C_2_ molecules, the formation of aromatics in Mo/H-SSZ-13 is partially inhibited. With a gradual decrease over time, C_6_H_6_ production is seen even after 90 min of reaction, while other authors report no aromatisation on Mo/H-SSZ-13 at such reaction times. This may be due to the differences arising in catalyst preparation; i.e., the formation of mesopores during calcination can favour the diffusion of aromatics in our catalyst during reaction. Initial H_2_ levels are comparable in both samples; the H_2_ production in Mo/H-SSZ-13 steeply decreases, evidencing a significantly faster deactivation. The thermogravimetric analysis (TGA) of the catalysts after 90 min of reaction show a higher carbon content for Mo/H-SSZ-13 (with 6.2 wt %) than for Mo/H-ZSM-5 (3.0 wt %), which points out the increased selectivity to carbon deposits in line with the observed rapid deactivation.

The derivative of the TGA curves (Figure 5b) shows that the carbon deposit burning off temperature is significantly higher for Mo/H-SSZ-13 than for Mo/H-ZSM-5 with maximum rates of combustion at 557 and 433 °C, respectively. This temperature difference cannot be attributed solely to the different carbon concentration found in the catalysts; Mo/H-SSZ-13 samples recovered after different MDA reaction times (7 to 90 min) with varying carbon deposit content (0 to 6.2 wt %) all exhibit comparatively higher carbon burning off temperatures with maximums of combustion rates >500 °C (Figure S7, Table S5).

The high carbon burning off temperature in Mo/H-SSZ-13 can be attributed to the location of carbon deposits—i.e., gas diffusion hindrance through small pores may delay the coke combustion inside the zeolite pores. Alternatively, Mo/H-SSZ-13 could lead to the formation of carbon deposits of a more stable nature. The Raman (Figure 5c) spectra of 90 min reacted catalyst do not reveal clear differences in the nature of carbon deposits. Both spectra exhibit the D4 band at 1200 cm^−1^ ascribed to either sp^2^-sp^3^ hybridised C-C and C=C stretching vibrations of polyenes [45], the D1 band ≈1360 cm^−1^ is attributed to in-plane breathing vibrations of sp^2^-bonded carbon, and the G band at 1611 cm^−1^ corresponds to in-plane stretching vibrations of pairs of sp^2^ C atoms. The position of the latter appears at higher wavenumbers than expected (generally reported ≈1580 cm^−1^) which can be attributed to the contribution from the D2 band (≈1620 cm^−1^) corresponding to edges of graphitic nanocrystallites [46,47]. This suggests the presence of very small carbon crystallites with a high number of edges [47,48] in Mo/H-SSZ-13 and Mo/H-ZSM-5. Furthermore, the intense D1 band is indicative of high structural disorder in carbon deposits of both samples.

**Figure 6:**
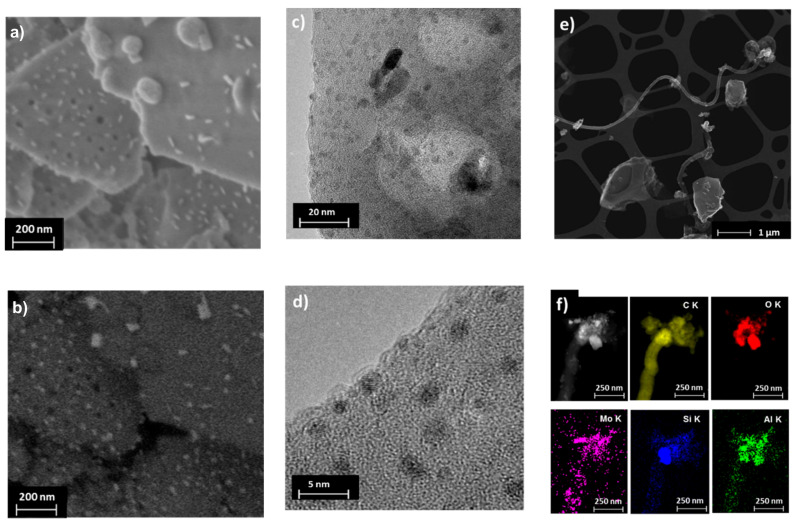
Microscopy images for Mo/H-SSZ-13 after 10 h of reaction. High-resolution SEM secondary (**a**) and backscattered (**b**) electron images; TEM images at two different magnifications (**c**,**d**); dark field TEM image of filamentous carbon (**e**); and the TEM-EDX elemental maps of filamentous carbon (**f**).

Microscopy images (Figure 6) of deactivated catalysts (after 10 h of MDA) reveal the presence of molybdenum-rich particles (3 to 50 nm). These particles seem to be smaller and more abundant than for the calcined Mo/H-SSZ-13, suggesting that they arise from the sintering and migration of MoC_x_ species during MDA reaction, as seen in the medium pore catalysts [20]. The TEM images also allow distinguishing a carbon deposit layer covering the Mo particles and the zeolite crystal surface (Figure 6d). Interestingly, TEM-EDX analysis of reacted Mo/H-SSZ-13 sample show the presence of large carbon nanotubes or nanofibers with a diameter of ≈120 nm and variable length of several microns (Figure 6e,f). Such carbon species were not observed in Mo/H-ZSM-5, although Hensen et al. have previously reported smaller nanotubes (≈20 nm) on 10 h MDA reacted Mo/H-ZSM-5 [31].

## 3. Summary and Conclusions

Mass spectrometry results revealed a decreased benzene formation on small pore Mo/H-SSZ-13 catalyst; besides, higher carbon deposit content was detected for the 90 min spent Mo/H-SSZ-13 (6.2 wt %) catalyst as compared with the widely investigated Mo/H-ZSM-5 (3.0 wt %). In line with previous reports [34], this indicates increased selectivity to carbon deposits for the small pore catalyst consistent with its faster deactivation. To gain insight into such a fast deactivation process, structural differences in both catalysts were evaluated by *operando* XAS experiments. In addition to the formation of Mo carbides during reaction, a reduction to metallic Mo was observed in Mo/H-SSZ-13, which has not been seen in Mo/H-ZSM-5 and other zeolite topologies. The consecutive reaction—regeneration cycles performed at different temperatures revealed that the degree of reduction increases with increasing MDA temperature.

Studies on the Mo/H-SSZ-13 calcination process imply that differences exist in the initial Mo distribution after calcination of the physical mixture of the zeolite and the MoO_3_ precursor. While SPD shows that a fraction of Mo migrates into the vicinity of the 8R sites of the CHA structure during calcination, part of the Mo is seen to remain outside the zeolite pores, as shown by the obtained electron densities. In agreement, electron microscopy reveals that the ion exchange process is less effective in the small pore system, as strong zeolite dealumination was observed leading to Al_2_(MoO_4_)_3_ particles formation on the H-SSZ-13 zeolite surface.

The characterisation of spent catalysts also indicates differences in both systems. The carbon burning off temperatures obtained by TGA for samples after 90 min of MDA imply that carbon deposits formed on small pore catalysts are more stable than in medium pores. Although no significant differences were seen in the Raman spectra, TEM images recorded for the 10 h reacted samples reveal the formation of large carbon nanotubes or fibres on Mo/H-SSZ-13.

Being able to tune the MDA product distribution by using different zeolite topologies would bring a new scope into the MDA reaction, especially if selectivity to light olefins was enhanced by the use of small pore zeolites. However, the very few MDA studies using small pore supports have reported poor catalytic performance, and further structural investigations are required to properly assess the potential of small pores in MDA. Or results shows that the low performance observed on small pore zeolites is driven by the difficulty of dispersing Mo inside the pores cavities and the subsequent effects (i.e., framework dealumination followed the concomitant formation of both mesopores and Al_2_(MoO_4_)_3_ particles). Hence, this work encourages the careful optimisation of the synthesis methods aiming for an improved Mo ion exchange process in small pore zeolites. In this regard, one-pot synthesis strategies combined with mild calcination could be considered. Obtaining comparable Mo dispersion as in medium pore zeolites will allow for an accurate assessment of the zeolite topology effects on MDA product distribution and to better understand the full potential of small pore zeolites as a selective catalyst toward light olefins. In addition, the results also call for further investigations on the causes of Mo reduction into the metallic state and its implications on the nature of carbon deposits formed under MDA. 

## 4. Materials and Methods 

*Synthesis:* The ZSM-5 (MFI structure) was purchased from Zeolyst (CBV3024E) in its ammonium form. The proton form was obtained by calcination in static air at 550 °C for 6 h, using a heating rate of 2 °C/min. H-SSZ-13 (CHA structure) was prepared in fluoride media with trimethyl-1-adamantamonium hydroxide as the structure directing agent following previously reported methods [49,50,51]. Mo/H-SSZ-13 and Mo/H-ZSM-5 catalysts were prepared by mechanically mixing MoO_3_ (Sigma Aldrich, 99.95%) powder and the zeolite in an agate mortar for 0.5 h. The resulting physical mixtures are named as as-prepared catalysts. Then, the samples were calcined in air at 700 °C for 30 min using a ramp of 5 °C/min.

*Synchrotron powder diffraction (SPD):* Data were collected at a BM01 beam line (the Swiss-Norwegian beamline) of the European Synchrotron Facility (ESRF).The diffractometer is based on a Huber goniometer with a Pilatus 2 M detector. X-rays with a wavelength of 0.69811 Å were used, which were selected by 2 Rh coated mirrors, as well as a silicon (111) double crystal monochromator. The beamline setup is described in detail elsewhere [52]. Data were collected at a sample to detector distance of 260 mm—calibrated using NIST SRM660b lanthanum hexaboride—and a 2-q range of 2 to 478 was used in the Rietveld analysis. As-prepared Mo/H-SSZ-13 was packed between plugs of quartz wool in 0.5 mm quartz capillaries and mounted in a Norby-type flow cell [53]. The samples were calcined by heating to a temperature of 600 °C with a heating rate of 6 °C/min and held for 8 h before cooling down to room temperature. A hot air blower was used to heat the sample, and calcination was done under 10 mL/min flow of 20% O_2_ in He (GHSV = 750 h^−1^). Data were collected with a collection time of 10 s per frame and converted to 1-D powder patterns using Fit2D [54] and the SNBL scaling software [55]. Rietveld and difference Fourier analysis was carried out with the TOPAS software (version 5), and the initial model for the framework was taken from the zeolite structure database; the MoO_3_ model was taken from the ICDD database. After refinement of the framework model to obtain reasonable starting parameters, difference Fourier maps were used to locate the Mo atoms. The scaling factor was obtained using the high-angle data, which are not significantly affected by adsorption of molecules in a zeolite framework [56]. This was fixed for determination of the difference maps using the whole powder pattern. In the final Rietveld refinements, all framework atom positions were refined without restrains along with isotropic thermal parameters for the silicon and oxygen atoms, background, peak broadening, scale factor, lattice parameters, zero point correction, and occupancies for the non-framework atoms. The same difference Fourier mapping method was used on a dataset collected below 100 °C to find the main positon for adsorbed water in the framework (coordinates: 0.49942, 0.24971, 0.76584), with MoO_3_ present as a secondary phase. The entire temperature series for heating from room temperature to 600 °C under flowing 20% oxygen/He was refined simultaneously using a parametric approach [57], starting with the basic model from the fit at 600 °C. An initial round of surface refinement (all parameters refined freely for all diffraction patterns except for zero error, which had a single value for the entire dataset) established that the disappearance of MoO_3_ from the mixture was extremely noisy, but it could be fitted with sigmoidal function (BoltzIV function in Origin 2020). Then, a parametric refinement was carried out in which the scale factors of the MoO_3_ phase were determined using the sigmoidal equation obtained from Origin:

(1) $$y=\left(\left(x-\mathrm{vrev}\right)*\mathrm{gmax}\right)/\left(1+\exp\left(\left(x-\mathrm{vhalf}\right)/\mathrm{dx}\right)\right)$$

with the coefficients vrev, gmax, vhalf, and dx refined in the least squares minimization. This reduced the number of parameters governing the MoO_3_ scale factors from 450 to 4. The smoothing of the MoO_3_ weight fraction led to improvements in the 8R occupancy noise levels (see Figure S2).

*X-ray absorption spectroscopy (XAS):* Studies were performed on a B18 beamline at Diamond Light Source [58] at Harwell Campus, United Kingdom. The storage beam energy was 3 GeV, and the ring current 300 mA. Mo K-edge spectra (in the range of 19,797 to 21,000 eV) were collected in transmission mode using ion chamber detectors with a fast scanning Si(111) double crystal monochromator, with a Mo foil placed between I_t_ and I_ref_. X-ray beam dimensions at the sample position were ca. 1 × 1 mm^2^. In situ experiments were performed using a setup developed by A. B. Kroner et al. [59]. Mo K-edge XAS spectra were collected under *operando* MDA conditions where the reaction products were detected by an online mass spectrometer (OmniStar GSD 320O1). The first experiment consisted of MDA studies using Mo/H-ZSM-5 and Mo/H-SSZ-13. In these measurements, the as-prepared samples (0.425–0.150 mm sieve fractions) were placed in 3 mm diameter quartz capillaries and calcined in situ at 700 °C for 30 min (20% O_2_ in He and heating ramp of 5 °C/min). After flushing with Ar for 15 min, 50% CH_4_/Ar mixture was flowed, and the MDA reaction was carried out at 700 °C for 90 min with a gas hour space velocity (GHSV) of 3000 h^−1^. In a second experiment, XAS data were collected on a Mo/H-SSZ-13 during *operando* MDA reaction—reactivation cycles at different temperatures. Regeneration was carried out by flowing 20% O_2_/He. The cycles consisted of the following experimental sequence:

Cycle 1, calc.: calcination of the as-prepared sample at 700 °C for 30 min (5 °C/min).

Cycle 1, MDA 650 °C: temperature was adjusted to 650 °C. Lines were flushed with Ar for 15 min; then, MDA was performed for 70 min at 650 °C.

Cycle 2, calc.: regeneration for 10 min at 650 °C.

Cycle 2, MDA 650 °C: after 15 min Ar flush, MDA was performed for 70 min at 650 °C.

Cycle 3, calc.: the temperature was increased to 780 °C followed by 10 min regeneration.

Cycle 3, MDA 780 °C: after 10 min Ar flush, MDA was performed for 70 min at 780 °C.

XAS data analysis was performed using the Demeter software package [60], and the range used for Fourier transform of the EXAFS was 3 < k < 9; all FT-EXAFS presented in this manuscript is non-phase corrected.

*Catalytic activity*: First, 0.6 g of as-prepared catalyst (150–425 µm sieve fraction) was introduced into a tubular quartz reactor (internal diameter = 0.7 mm). The sample was fixed in the isothermal zone of the oven by quartz wool. A total gas flow of 30 mL/min was fed by means of mass flow controllers (GHSV = 1500 h^−1^). The as-prepared physical mixtures were first calcined under 20% O_2_/He flow by heating up to 700 °C for 30 min using a heating rate of 5 °C/min. After flowing pure Ar for 30 min, a methane dehydroaromatisation reaction was initiated by flowing 50% CH_4_/Ar. The reaction products were analysed by an online mass spectrometer (OmniStar GSD 320O1). All MS data intensity presented in this manuscript are normalised to the Ar signal and are plotted in logarithmic scale.

*Scanning electron microscopy (SEM)* analysis was done in a Zeiss ultra 55 Field emission electron microscope. Compositional analysis and low-resolution general imaging were carried out with accelerating voltage of 20 kV, 30–60 micron aperture, and 7–8 mm working distance. High-resolution images were also taken with an accelerating voltage of 1.6 kV, 20–30 micron aperture, and 2–3 mm working distance.

*Transmission Electron Microscopy* images (TEM) were taken in an JEM 2800 (Scanning); voltage was 200 kV and the aperture was 70 and 40 µm. Bright-field imaging mode was done using a charge-coupled device CCD camera in high magnification, while dark-field (Z-contrast) imaging was carried out in scanning mode using an off-axis annular detector. The secondary electron signal was acquired simultaneously with the other TEM images providing topological information of the sample. Compositional analysis was performed by X-ray emission detection (EDX) in the scanning mode.

*Thermogravimetric* measurements were carried out in a TA Q50 instrument; all samples were heated up to 950 °C using a temperature ramp of 5 °C/min under an air flow of 60 mL/min and held at 950 °C for 5 min.

*Kerr-gated Raman* spectroscopy measurements were carried out in the Ultra setup in the Central Laster Facility to study the nature of carbonaceous deposits on reacted catalysts. The measurements were carried out using 400 nm Raman probe beam. Then, an 800 nm pulsed laser beam was used to activate the CS_2_ Kerr gate. Toluene impregnated H-ZSM-5 was used for Raman shift calibration.

*Elemental analysis* was carried out using inductively coupled plasma optical emission spectroscopy (ICP-OES) in a Perkin Elmer Optical Emission Spectrometer Optima 3300 RL.

## Figures and Tables

**Table 1:** Oxidation state, Mo K-edge energies, and edge step values for Mo/H-SSZ-13 after calcination and 70 min of MDA at different temperatures.

Experiment	Stage of the Cycle	Oxidation Estate	Edge Position (eV)	Edge Step
Exp. 1	As-prepared	5.9	20,014.6	0.360
	Calcined	5.8	20,014.3	0.158
	MDA 700 °C	2.1	20,007.4	0.161
Exp. 2	As-prepared	5.8	20,014.4	0.410
	Cycle 1, calc.	5.8	20,014.5	0.194
	Cycle 1, MDA 650 °C	2.4	20,008.1	0.197
	Cycle 2, calc.	5.8	20,014.3	0.180
	Cycle 2, MDA 650 °C	2.5	20,008.2	0.178
	Cycle 3, calc.	5.8	20,014.3	0.158
	Cycle 3, MDA 780 °C	1.8	20,006.9	0.157

## Supplementary Materials

The following are available online. Figure S1. Rietveld fit for Mo-SSZ-13 using Mo positions established by difference Fourier mapping. The inset shows the quality of the fit at high angle. Table S1. Rietveld refinement details for calcined (in situ, 600 °C, 8 h, air) Mo-SSZ-13. The refinement was done with TOPAS 5. Table S2. Atomic coordinates for calcined (in situ, 600 °C, 8 h, air) Mo-SSZ-13. Table S3. Bond lengths (Å) for calcined (in situ, 600 °C, 8 h, air) Mo-SSZ-13. Table S4. Bond Angles (Degrees) for calcined (in situ, 600 °C, 8 h, air) Mo-SSZ-13. Figure S2. Improvement of noise levels in MoO_3_ weight percentage (a) and 8R occupancy between surface and parametric refinement of the calcination data series (b). Figure S3. TEM microscopy for calcined Mo/H-SSZ-13 (700 °C in air, 30 min); a) dark-field image with the corresponding EDX maps, and b) EDX spectra at different regions of zeolite crystal. Figure S4. Data collected during operando MDA (700 °C, 50 % CH_4_/Ar, 3000 h^−1^, 90 min) studies on Mo/zeolites: Mo K-edge XANES spectra for Mo/H-SSZ-13 (a) and Mo/H-ZSM-5 (b); and mass traces recorded by MS for Mo/H-SSZ-13 (c) and Mo/H-ZSM-5 (d). Figure S5. MS data under methane dehydroaromatisation reaction products collected for Mo/H-SSZ-13 (a) and Mo/A-ZSM-5 (b) (50 % CH_4_/Ar, 1500 h^−1^, 700 °C, 90 min); the data is shown in logarithmic scale and all signals are normalised to the carrier gas signal (Ar). Figure S6. Mo K-edge EXAFS spectra showing: (a) calcined Mo/zeolite compared to Al_2_(MoO_4_)_3_; (b) Mo/zeolites after 90 min of MDA compared to Mo_2_C and metallic Mo; and (c) M Mo/H-SSZ-13 after 70 min of MDA reaction at 650 (cycle 1), 700 and 780 °C (cycle 3) compared to Mo and Mo_2_C references. Figure S7. Derivative of the TGA curves for Mo/zeolite catalysts recovered after different MDA reaction times. Solid line corresponds to Mo/H-SSZ-13 and dotted line to Mo/H-ZSM-5. Table S5. ICP results for Mo/HZSM-5 and Mo/H-SSZ-13 after calcination at 700 °C, as well as the TGA results for Mo/H-SSZ-13 reacted at different MDA reaction times (50% CH_4_/Ar, 1500 h^−1^, 700 °C).
