# Supplementary material for: Understanding the Deactivation Phenomena of Small-Pore Mo/H-SSZ-13 during Methane Dehydroaromatisation
Source: Molecules. 2020 Oct 30;25(21):5048. doi: 10.3390/molecules25215048 (PMC7663607; doi:10.3390/molecules25215048)
Supplement: Supplementary file 1 [file molecules-25-05048-s001.pdf]

molecules

Supporting Information

Understanding the deactivation phenomena of small-pore Mo/H-SSZ-13 during methane dehydroaromatisation

Miren Agote-Arán [1,2], Anna B. Kroner [2], David S. Wragg [3], Wojciech A. Sławiński [3,4], Martha Briceno [5], Husn U. Islam [5], Igor V. Sazanovich [6], María E. Rivas [5], Andrew W. J. Smith [5], Paul Collier [5], Inés Lezcano-González [1,7,*] and Andrew M. Beale [1,7,*]

Synchrotron powder diffraction analysis results are gathered in the following tables. The observed, calculated, and difference patterns obtained from Rietveld refinement are given in Table S1. The experimental parameters and goodness-of-fit factors, coordinates, and selected bond distance are shown in Table S2.

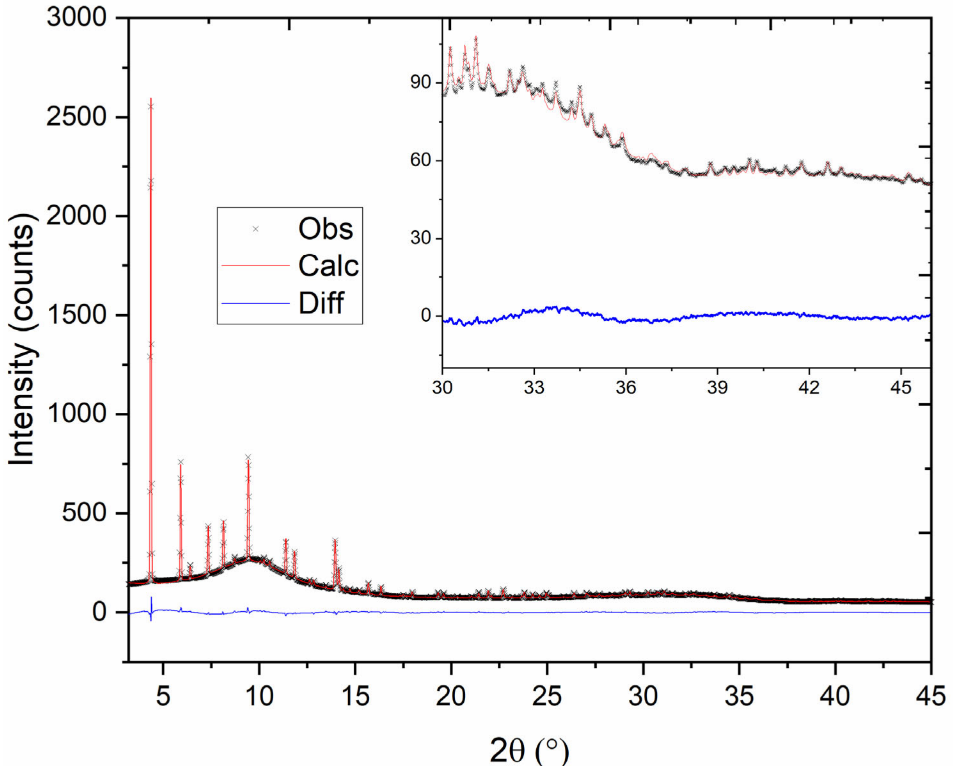

Figure S1. Rietveld fit for Mo-SSZ-13 using Mo positions established by difference Fourier mapping. The inset shows the quality of the fit at high angle.

Table S1. Rietveld refinement details for calcined (in situ, 600 °C, 8 h, air) Mo-SSZ-13. The refinement was done with TOPAS 5.

Parameter	Calcined sample (in situ)
Radiation, Wavelength (Å)	SNBL beamline BM01A, 0.69811
Crystal System	Rhombohedral (hexagonal axes)
Space Group	$R\bar{3}m$
Temperature	600 °C
a, c (Å), volume (Å3)	13.5367(4), 14.7522(6), 2341.1(2)
Rwp, Rp, Rexp	1.25, 1.59, 0.26
Rwp, Rp, Rexp - background	6.00, 4.47, 1.33
R Bragg	0.7
GooF	4.5
Parameters, restraints	37, 0
Number of Data Points	2721

Table S2. Atomic coordinates for calcined (in situ, 600 °C, 8 h, air) Mo-SSZ-13.

Atom	x	y	z	U(eq) [Å2]
Mo_8R	0	1/2	0	0.0127

Si1	0.0011(4)	0.2289(3)	0.10329(19)	0.0114(6)
O1	0.9008(4)	0.0992(4)	0.1230(7)	0.0295(15)
O2	0.6424(7)	0.6424(7)	1/2	0.0295(15)
O3	0.1180(4)	0.2359(9)	0.1303(7)	0.0295(15)
O4	0	0.2639(6)	0	0.0295(15)
Atom	x	y	z	U(eq) [Å2]

Table S3. Bond lengths (Å) for calcined (in situ, 600 °C, 8 h, air) Mo-SSZ-13.

Si1-Mo_D6R	3.189(5)	Si1-O2_g	1.595(9)
O1-Mo_D6R	2.564(10)	Si1-O3	1.588(10)
O3-Mo_D6R	3.008(10)	Si1-O4	1.598(4)
Si1-Mo_D6R	3.189(5)	Si1-O1_a	1.621(6)
O4-Mo_8R	3.196(9)		

Table S4. Bond Angles (Degrees) for calcined (in situ, 600 °C, 8 h, air) Mo-SSZ-13.

O3-Si1-O4	112.2(5)	O1-Si1-O2	106.5(5)
O1-Si1-O3	106.8(5)	Si1-O1-Si1	147.7(6)
O2-Si1-O3	111.1(5)	Si1-O2-Si1	153.1(7)
O1-Si1-O4	111.4(5)	Si1-O3-Si1	150.3(7)
O2-Si1-O4	108.7(4)	Si1-O4-Si1	145.0(5)
O1-Mo_D6R-O3	72.7(3)	Si1-Mo_D6R-Si1	52.5(1)

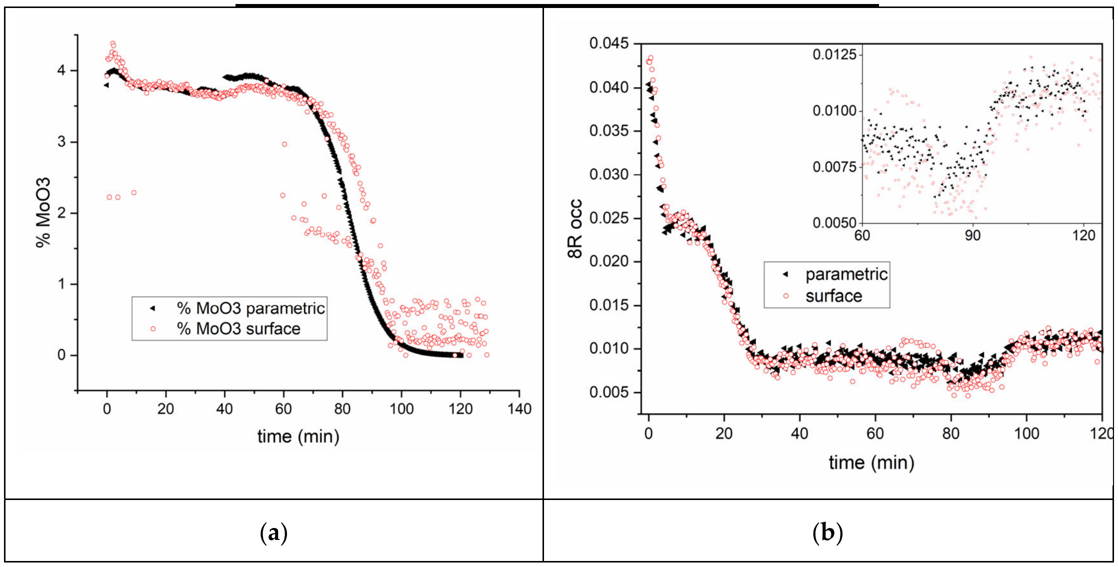

(**a**) (**b**)

Figure S2. Improvement of noise levels in MoO_3 weight percentage (**a**) and 8R occupancy between surface and parametric refinement of the calcination data series (**b**).

TEM-EDX analysis of Mo/H-SSZ-13 after calcination in air (700 °C, 30 min).

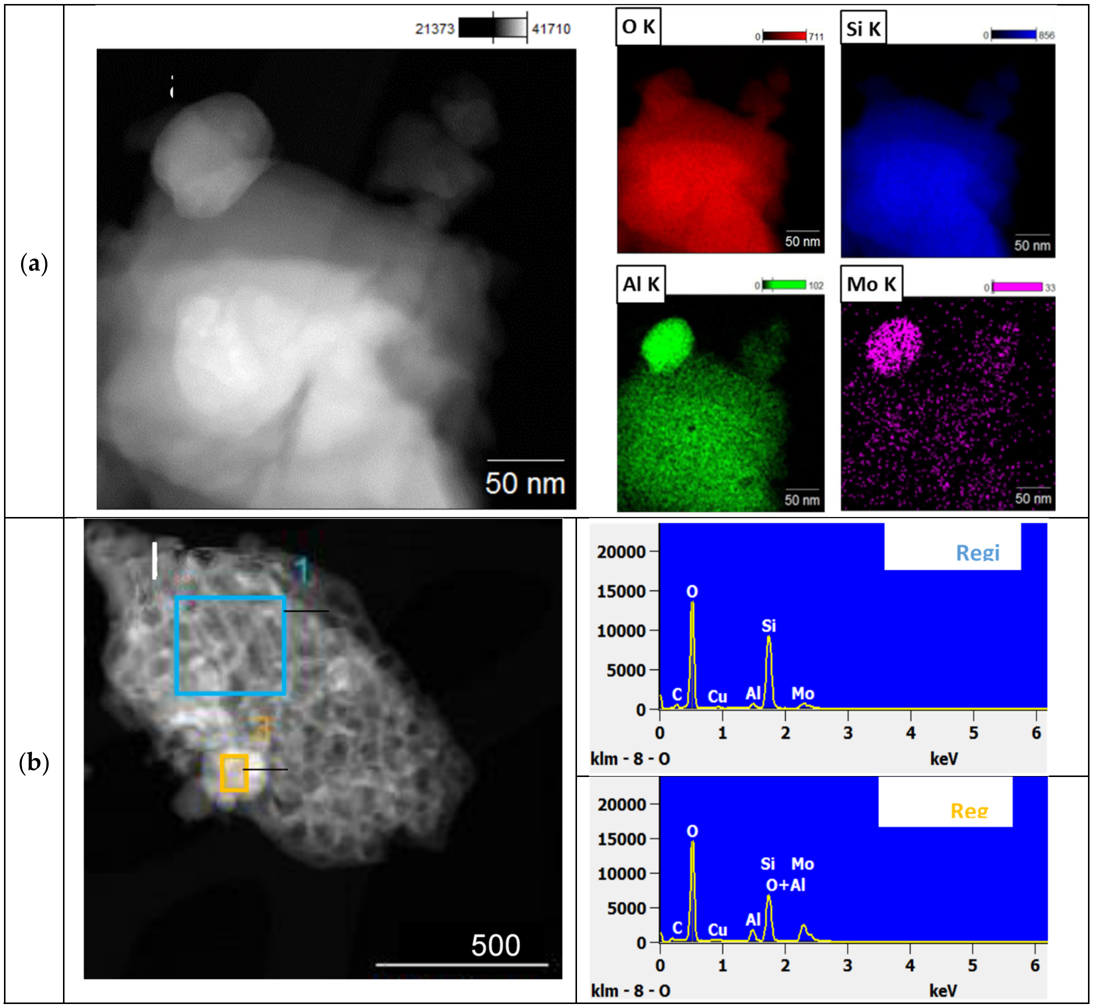

Figure S3. TEM microscopy for calcined Mo/H-SSZ-13 (700 °C in air, 30 min); a) dark-field image with the corresponding EDX maps, and b) EDX spectra at different regions of zeolite crystal.

X-ray absorption spectroscopy and mass spectrometry data obtained during *operando MDA* investigations for Mo/H-SSZ-13 and Mo/H-ZSM-5.

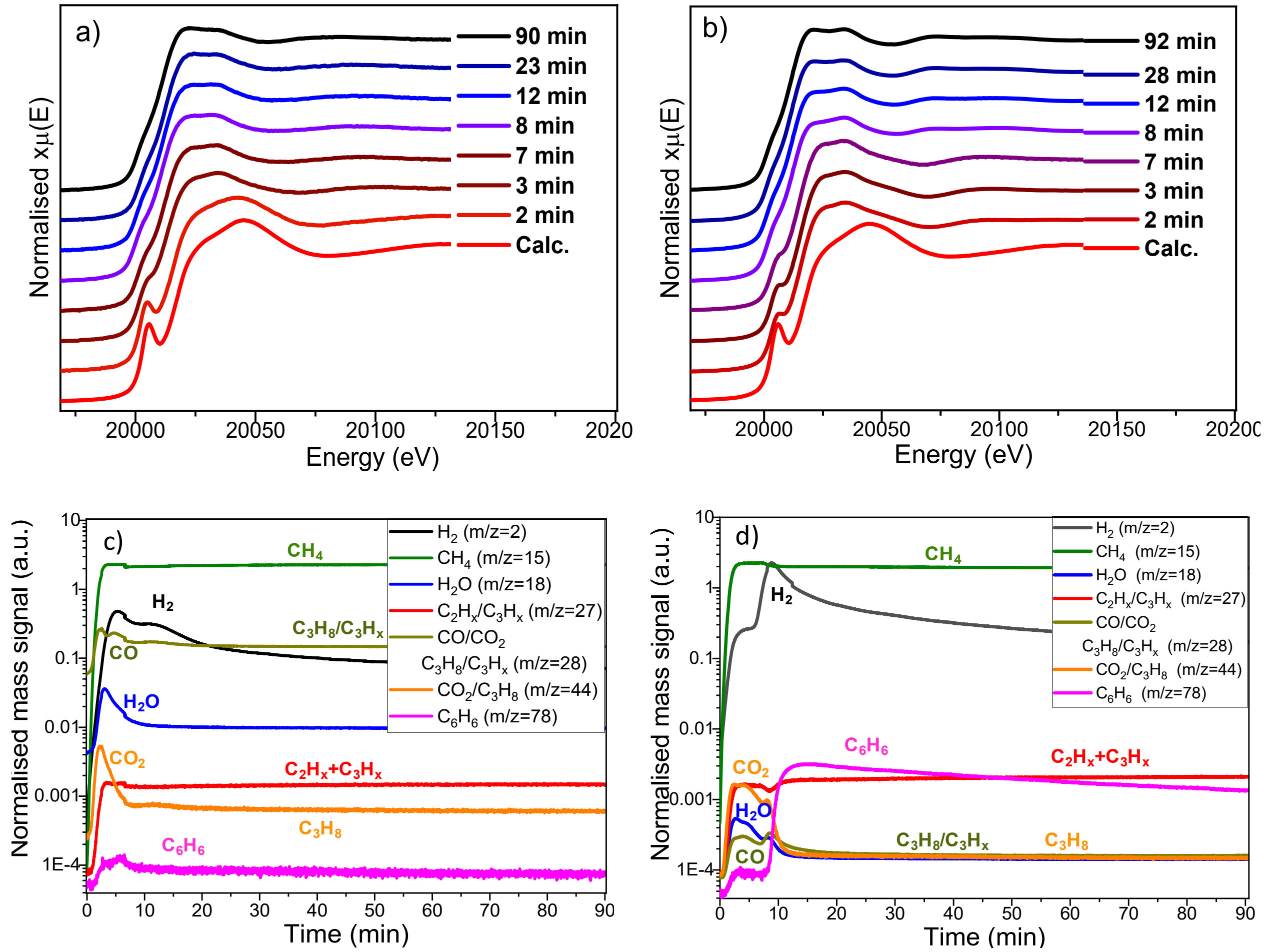

Figure S4. Data collected during operando MDA (700 °C, 50 % CH_4/Ar, 3000 h^{-1}, 90 min) studies on Mo/zeolites: Mo K-edge XANES spectra for Mo/H-SSZ-13 (a) and Mo/H-ZSM-5 (b); and mass traces recorded by MS for Mo/H-SSZ-13 (c) and Mo/H-ZSM-5 (d).

Mass trends of MDA reactions performed in the laboratory (50 % CH_4/Ar, 1500 h^{-1}, 700 °C) for Mo/H-SSZ-13 and Mo/H-ZSM-5.

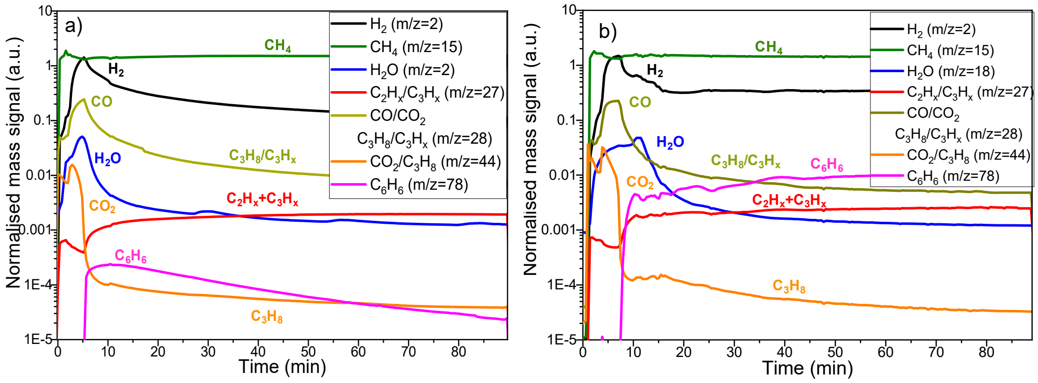

Figure S5. MS data under methane dehydroaromatisation reaction products collected for Mo/H-SSZ-13 (a) and Mo/A-ZSM-5 (b) (50 % CH_4/Ar, 1500 h^{-1}, 700 °C, 90 min); the data is shown in logarithmic scale and all signals are normalised to the carrier gas signal (Ar).

Mo k-edge spectra for Mo/zeolites under different reaction conditions as well as for Mo, Mo_2C and $Mo_2Al_3O_{12}$ references.

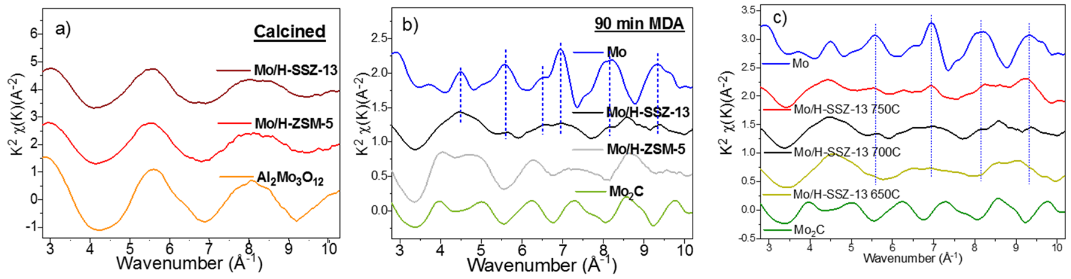

Figure S6. Mo K-edge EXAFS spectra showing: a) calcined Mo/zeolite compared to $Al_2(MoO_4)_3$; b) Mo/zeolites after 90 min of MDA compared to Mo_2C and metallic Mo; and c) M Mo/H-SSZ-13 after 70 min of MDA reaction at 650 (cycle 1), 700 and 780 °C (cycle 3) compared to Mo and Mo_2C references.

Themogravimetric analysis results for Mo/zeolites after different MDA reaction times (50 % CH_4/Ar, 1500 h^{-1}, 700 °C).

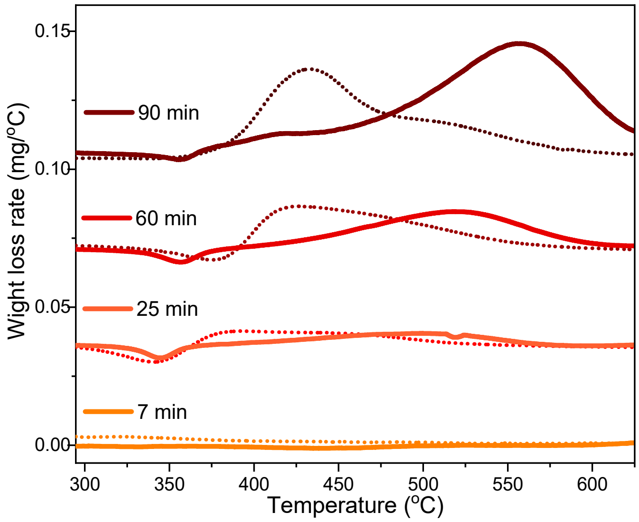

Figure S7. Derivative of the TGA curves for Mo/zeolite catalysts recovered after different MDA reaction times. Solid line corresponds to Mo/H-SSZ-13 and dotted line to Mo/H-ZSM-5.

Table S5. ICP results for Mo/HZSM-5 and Mo/H-SSZ-13 after calcination at 700 °C, as well as the TGA results for Mo/H-SSZ-13 reacted at different MDA reaction times (50% CH_4/Ar, 1500 h^{-1}, 700 °C).

Sample	Mo contents (wt. %)	Si/Al	Carbon content (wt. %)
Mo/H-SSZ-5 calc.	3.8	16.8	-
Mo/H-SSZ-13 calc.	3.9	14.0	-
Mo/H-SSZ-13 7 min	-	-	0.00
Mo/H-SSZ-13 25 min	-	-	0.80
Mo/H-SSZ13 60 min	-	-	1.84
Mo/H-SSZ-13 90 min	-	-	6.15
